# Brain Abnormalities in Schizophrenia: A Comparative Imagistic Study

**DOI:** 10.3390/medicina60040564

**Published:** 2024-03-29

**Authors:** Paula Simina Petric, Petru Ifteni, Ana Aliana Miron, Gabriela Sechel, Andreea Teodorescu

**Affiliations:** 1 Facultatea de Medicină, Universitatea Transilvania din Brașov, Bulevardul Eroilor 29, 500036 Brașov, Romania; paula_petric@yahoo.com (P.S.P.); aliana_mioc@yahoo.com (A.A.M.); gabisechel@yahoo.com (G.S.); andre_martie@yahoo.com (A.T.); 2 Spitalul Clinic de Psihiatrie și Neurologie Brașov, Str. Prundului No. 7-9, 500123 Brașov, Romania

**Keywords:** neuroimaging, first psychotic episode, schizophrenia, cerebral CT (computed tomography), cerebral density

## Abstract

*Background and Objectives*: Neuroimaging reveals a link between psychiatric conditions and brain structural–functional changes, prompting a paradigm shift in viewing schizophrenia as a neurodevelopmental disorder. This study aims to identify and compare structural brain changes found during the first schizophrenia episode with those found after more than 5 years of illness. *Materials and Methods*: This prospective study involved 149 participants enrolled between 1 January 2019 and 31 December 2021. The participants were categorized into three groups: the first comprises 51 individuals with an initial psychotic episode, the second consists of 49 patients diagnosed with schizophrenia for over 5 years, and a control group comprising 50 individuals without a diagnosis of schizophrenia or any other psychotic disorder. All participants underwent brain CT examinations. *Results*: The study examined all three groups: first-episode schizophrenia (FES), schizophrenia (SCZ), and the control group. The FES group had a mean age of 26.35 years and a mean duration of illness of 1.2 years. The SCZ group, with a mean age of 40.08 years, had been diagnosed with schizophrenia for an average of 15.12 years. The control group, with a mean age of 34.60 years, had no schizophrenia diagnosis. Structural measurements revealed widening of frontal horns and lateral ventricles in the SCZ group compared to FES and the FES group compared to the control group. Differences in the dimensions of the third ventricle were noted between SCZ and FES, while no distinction was observed between FES and the control group. The fourth ventricle had similar measurements in FES and SCZ groups, both exceeding those of the control group. Our results showed higher densities in the frontal lobe in schizophrenia patients compared to FES and the control group, with the control group consistently displaying the lowest densities. *Conclusions*: In summary, our comparative imaging analysis of schizophrenia patients, first-episode schizophrenia, and control patients revealed distinct ventricular patterns, with SCZ showing greater widening than FES and FES wider than the control group. Frontal lobe density, assessed via cerebral CT scans, indicated a higher density in the SCZ group in both anterior and posterior cortex portions compared to FES and the control group, while the left posterior cortex in FES had the highest density. These findings highlight unique neuroanatomical features across groups, shedding light on structural differences associated with different stages of schizophrenia.

## 1. Introduction

Studies in neuroimaging have shown that psychiatric conditions are associated with both structural and functional brain changes. Although the subject is of great interest, there is limited information regarding the etiopathogenesis of schizophrenia or other psychotic disorders. Early studies were based on post-mortem brain examinations that did not provide concrete information due to the aging process, chronicity of the illness, and medication effects. Studies conducted on patients in the early stages of the disease, especially those in the first psychotic episode, do not have such limitations [1,2,3].

In more recent years, the idea that schizophrenia could be a neurodevelopmental disorder has been increasingly emphasized. Brain development begins during the intrauterine life and continues until adulthood. Early brain development (the pre- and perinatal period) is characterized by neurogenesis and neuronal migration, followed by abundant synapse proliferation that continues into childhood. This is followed, during the later phases of brain development, by the elimination or programmed removal of redundant synapses (in late childhood and adolescence) and myelination (which continues into adulthood). Synaptic and neuronal losses also occur later in life, due to aging [4,5,6]. Several physiopathological models have been described to explain the underlying brain mechanisms in schizophrenia. The first model is that of early development, suggesting that anomalies in brain development during the prenatal period mediate the failure of brain functions in young adulthood. This hypothesis is supported by several findings, such as increased rates of birth complications, minor physical anomalies, mild neurological signs, and minimal behavioral abnormalities in children who later develop schizophrenia [7,8]. It has been suggested that neuronal migration, which mainly occurs during the second trimester of fetal development, may be defective [9]. Animal models have suggested that neonatal lesions of the hippocampus can lead to a reduction in dopamine transporter messenger RNA (mRNA) [10]. Other models have proposed that a developmental disorder of the hippocampus leads to abnormal limitation of limbic system activity, resulting in valence-based response strategies (i.e., amygdala-related) as opposed to goal-directed response strategies (i.e., prefrontal-related) [11].

The onset of prominent symptoms of schizophrenia during adolescence or young adulthood raises intriguing questions about the potential role of developmental factors preceding or coinciding with the emergence of psychosis. This critical period of brain maturation is characterized by intricate neurodevelopmental processes that, if disrupted, might contribute to the genesis of psychiatric disorders.

One plausible mechanism involves the programmed elimination of synapses, a pivotal aspect of neural development. If this elimination process becomes overly aggressive, it has the potential to trigger a significant loss of synapses within the glutamatergic system. Such synaptic alterations, particularly within the intricate web of glutamatergic pathways, are posited to be instrumental in precipitating the onset of schizophrenia. This hypothesis gained support from post-mortem studies conducted on individuals with schizophrenia, revealing noteworthy reductions in dendritic density within cortical brain regions—an observation that aligns with the concept of aberrant synaptic dynamics as a precursor to the disorder [12].

Moreover, the declining trajectory witnessed in certain patients during the initial years of schizophrenia lends support to the idea of a degenerative process that unfolds after the initial onset. This process is theorized to involve the gradual loss of pivotal neuronal or glial elements, entailing a dynamic interplay between anomalies in neurodevelopment and the ongoing cascade of degenerative changes. The delicate equilibrium involved in the formation and elimination of synapses, crucial for sculpting the neural circuitry, may experience dysregulation, thereby playing a contributory role in the observed deterioration [13].

The initial documentation of ventricular enlargement in schizophrenia emerged through the utilization of pneumoencephalography. In this pioneering study, not only was the ventricular enlargement noted, but there was also a distinct observation of variations in density and surface area between the two cerebral hemispheres [6]. This groundbreaking research laid the foundation for subsequent investigations that not only validated the asymmetry between the hemispheres but also underscored a more pronounced ventricular enlargement in individuals grappling with chronic schizophrenia, as opposed to those in the early phases of the disorder. These subsequent studies reinforced the understanding that structural alterations within the brain, particularly involving the ventricles, are dynamic and evolve throughout the course of schizophrenia. The observed asymmetry in the hemispheres and the intensified ventricular enlargement in chronic cases highlight the progressive nature of these changes. This suggests that the anatomical disruptions within the brain, as reflected in ventricular size and cerebral asymmetry, tend to intensify in parallel with the chronicity of schizophrenia. In essence, the evolution of our understanding from the initial descriptions of ventricular enlargement to the recognition of hemispheric asymmetry and exacerbated ventricular changes in chronic stages paints a comprehensive picture of the structural dynamics associated with schizophrenia [14,15].

Studies based on computer tomography images have demonstrated that, when variables (such as age, and duration of illness) are controlled, the age of onset of the disease predicts various aspects related to the subsequent course of the disease [16]. Findings observed with CT scanning include ventricular enlargement and cortical atrophy (especially in the frontal lobes) and are described in patients with chronic schizophrenia. Previous reports show that ventricular size increases with disease progression [17]. Further research has found that volumes of the temporal lobe in patients with schizophrenia are smaller compared to control subjects. The superior temporal gyrus (which is part of Wernicke’s area) is also reduced in volume, which could eventually explain the catatonic behavior and language disorders observed in certain patients with schizophrenia. Some studies have also identified atrophy of the parietal lobe (especially the cingulate gyrus and supramarginal gyrus) and atrophy of the occipital lobe; although, these are not common and are often present in the later stages of the disease [18,19].

Environmental factors, such as substance abuse and psychosocial stress, may also be potential secondary triggers accompanying the onset and progression of the disease. Neurobiological studies of early onset schizophrenia, especially neuroimaging studies, have the potential to examine predictions generated by these seemingly contrasting models.

The objective of this study was to evaluate the presence of structural brain changes in first-episode schizophrenia through imaging investigations and then compare the findings with the results of patients with chronic schizophrenia and with individuals without schizophrenia or neurological disease.

## 2. Methods

### 2.1. Study Design

This was a prospective study conducted in the Clinical Hospital of Psychiatry and Neurology Brașov, Romania, a medical academic facility with 160 beds for acute psychiatric hospitalizations and 315 beds for long-term psychiatric hospitalizations. The enrollment period was from 1 January 2019 to 31 December 2021. In total, 149 patients were enrolled in the study. This project was approved by the local Ethics Committee (Approval No. 6 from 18 December 2018) and was part of a doctoral research project.

### 2.2. Study Population

Participants were divided into three groups: a group of 51 patients experiencing their first psychotic episode, a group of 49 patients diagnosed with schizophrenia for more than 5 years, and a control group consisting of 50 patients without a diagnosis of schizophrenia or any other psychotic or neurological disorders.

### 2.3. Inclusion and Exclusion Criteria

Inclusion criteria for study groups were age between 18 and 45 years, diagnosis of first-episode schizophrenia and schizophrenia according to DSM-5 criteria and undergoing antipsychotic treatment. All patients were voluntarily hospitalized and signed informed consent to participate in the study. Exclusion criteria comprised psychiatric conditions other than schizophrenia or acute psychotic disorder, age below 18 or above 45 years, patients not receiving antipsychotic treatment, history of neurological conditions, history of organic brain disorders, alcohol or drug abuse, history of cranio-cerebral trauma, and refusal to undergo CT exploration.

### 2.4. Data Collected

**Figure 1:**
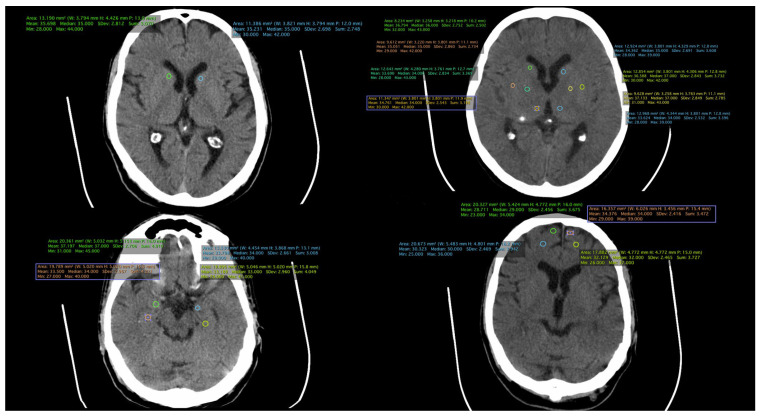
CT scan of a patient from the FES group.

**Figure 2:**
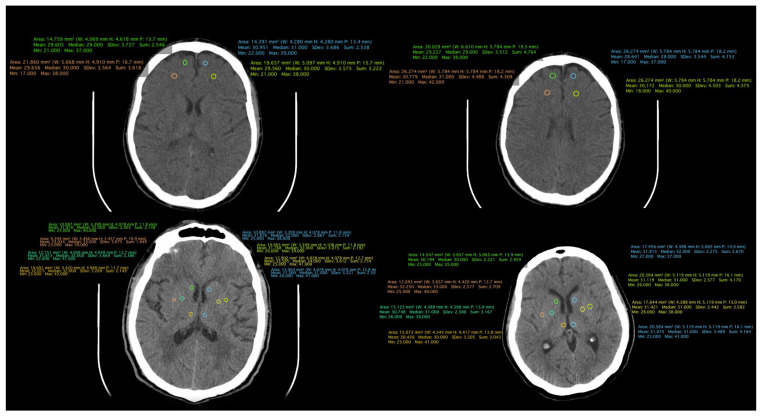
CT scan of a patient from the schizophrenia group.

**Figure 3:**
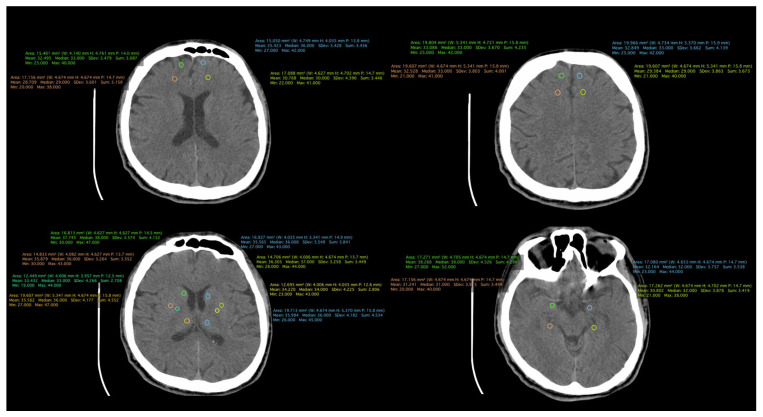
CT scan of a patient from the control group.

All patient-related information was collected from the paper and electronic patient records. Data were collected by certified medical staff, including board-certified psychiatrists and an imaging specialist. The database included demographic data, information regarding the onset and duration of the illness, the number of episodes, and the type of treatment received. Additionally, information obtained through CT examinations was incorporated, including measurements such as the transverse diameter between the frontal horn tips on axial section, transverse diameter at the level of the lateral ventricle bifurcation at the most cranial point of the ventricle on axial section, transverse diameter at the level of the diencephalic third ventricle on axial section, antero-posterior diameter at the level of the fourth ventricle on axial section, and density measurements in both the anterior and posterior portions of the frontal lobe at the cortical and subcortical levels. (Figure 1, Figure 2 and Figure 3).

CT examinations were performed using the Somatom Spirit apparatus, identification number 10165566, class IIb, which records at a frame frequency of at least 10 frames per second, resulting in 1100 images per examination. The resolution is 15.5 lp/cm.

### 2.5. Study Flowchart

**Figure 4:**
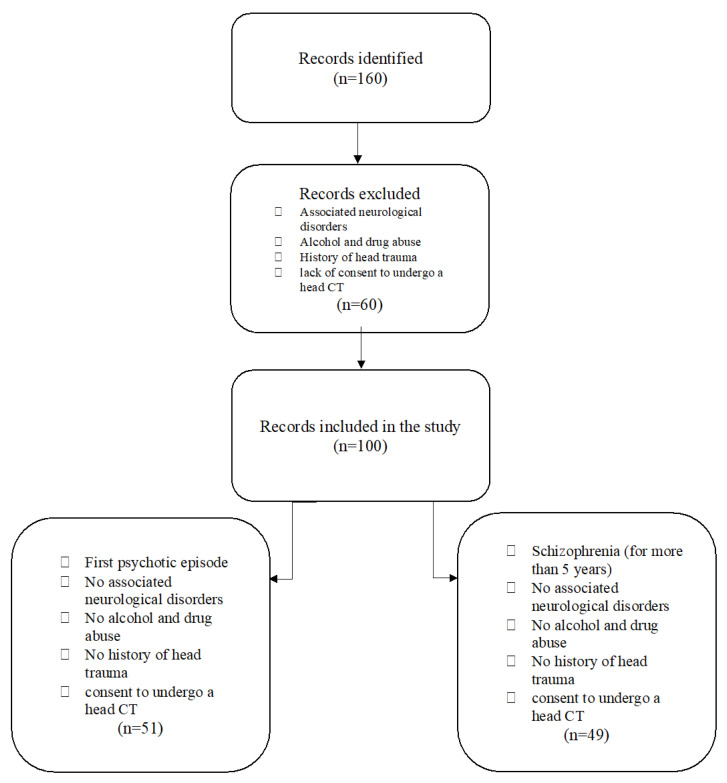
Study flowchart.

A total of 160 patients with a psychiatric diagnosis were assessed; 60 patients were excluded from the study for not meeting all the inclusion criteria. Finally, 100 patients were enrolled; they were divided into two analysis groups: one with 51 patients diagnosed with acute psychotic disorder and another with 49 patients diagnosed with schizophrenia. The control group consisted of 50 participants aged between 18 and 45 years, without psychiatric diagnoses, neurological disorders, organic brain disorders, alcohol or drug abuse, or a history of cranio-cerebral trauma. Control group participants provided informed consent to participate in the study and expressed their agreement to undergo cerebral CT (Figure 4).

### 2.6. Statistical Analysis

The statistical analysis was comprehensive and utilized various tests to rigorously evaluate the study data. The F-test, applied to assess the equality of variances among different groups, served as an initial exploration of variability. Subsequently, independent samples *t*-tests were employed for comparisons between two groups, providing valuable insights into specific contrasts within the data. For analyses involving three or more groups, analysis of variance (ANOVA) was utilized, allowing the examination of mean differences. When ANOVA results indicated significance, post hoc tests were conducted to pinpoint specific group variations. All statistical tests were executed at a predetermined significance level of 0.05. The statistical software SPSS version 20.00 facilitated the execution of these analyses, providing a robust foundation for interpreting the observed differences and relationships within the dataset and ensuring the reliability of study findings.

## 3. Results

Of the 160 patients initially identified as eligible for the study, after applying the inclusion and exclusion criteria, 60 patients were eliminated. The remaining 100 patients were divided into two groups: the FES group, which included 51 patients experiencing their first psychotic episode, and the SCZ group, consisting of 49 patients with schizophrenia and a disease duration of more than 5 years. The control group (Control) was composed of 50 subjects without any psychiatric illness.

Regarding the demographic data of the groups, in the FES group, the mean age was 26.35 years, and the mean age of onset was around 25 years (Table 1). The percentage of male patients was 54.9%, and all patients were experiencing their first episode of the illness, with a mean duration of illness of 1.2 years. In the SCZ group, the mean age was 40.08 years, and the mean age of onset was around 25 years. The mean duration of illness was 15.12 years, and the percentage of male patients was 48.98%. In the control group, the mean age of participants was 34.60 years, with a percentage of male patients of 68%.

**Figure 5:**
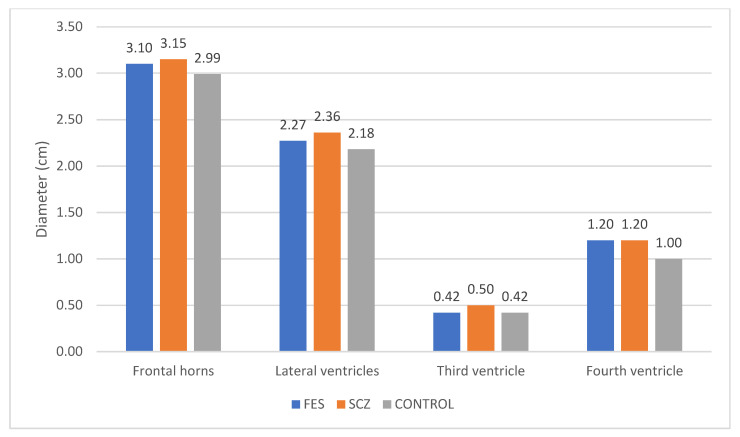
Variations in diameter among the three groups concerning the frontal horns and cerebral ventricles.

The frontal horns, lateral ventricles, third ventricle, and fourth ventricle were measured in patients from all three groups. A greater widening of the frontal horns and lateral ventricles was observed in SCZ patients compared to FES, and similarly, FES patients compared to the Control group. Regarding the third ventricle, we noted a difference in the SCZ group compared to FES, but no difference was observed between FES and the control group. As for the fourth ventricle, the average values were the same for the FES and SCZ groups, both of which were higher than the Control group (Figure 5).

**Figure 6:**
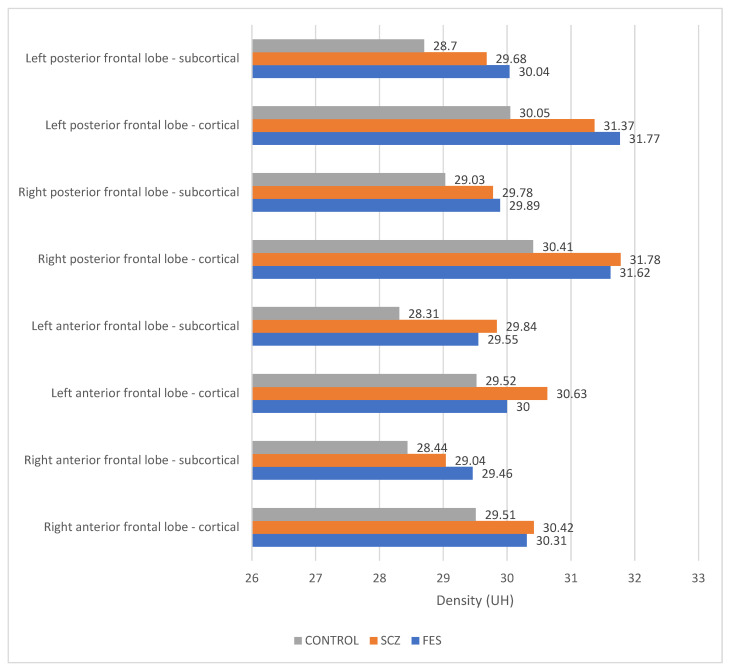
Densities across frontal lobe structures.

The densities of structures in the frontal lobe were measured using cerebral CT explorations. In the cortical region, a higher density is evident in both the anterior right and left portions, as well as in the posterior right portion, in patients with schizophrenia compared to those in the first episode of the illness and those in the control group. As per the cortical area of the left posterior region, the highest density is observed in patients in the FES group. Notably, in the subcortical portion, a higher density is recorded in the anterior left region of the frontal lobe in schizophrenia patients, with the highest densities observed across all other regions in the FES group. The lowest densities across all measured structures were consistently found in the control group (Figure 6). The statistical analysis using the ANOVA test for each structure individually is described in Table 2.

We considered the null hypothesis ($H_{0})$ and the alternative hypothesis $(H_{1}$) as follows:

**Hypothesis** **0 (H0).** *There is no significant difference between groups in terms of cerebral nervous tissue density*.

**Hypothesis** **1 (H1).** *There is a significant difference between groups in terms of cerebral nervous tissue density*.

In the ANOVA analysis, we noted that the null hypothesis is excluded for all situations except for the frontal left anterior cortical and frontal right anterior cortical regions, respectively. In all other situations, there is a statistically significant difference in brain density between groups.

Performing the *t*-test for independent samples, the groups were compared two by two. The results are detailed in Table 3.

In all statistically significant situations, the density of nervous tissue is constantly highest for the SCZ group (schizophrenia patients having multiple psychotic episodes) in comparison with the FES group (patients with first psychotic episode) and the Control group (healthy subjects). We noted that in these cases, the density of nervous tissue decreases as follows:

SCZ Group > FES Group > Control Group

In Table 4 we present a commonly used measure of the effect size in the independent samples t-test is the Bravais–Pearson correlation coefficient *r*.

The values can be interpreted as follows:

An *r* < 0.3 indicates a small effect;
An *r* in the range between 0.3 and <0.5 indicates a medium effect;
An *r* in the range of 0.5 to 1.0 indicates a large effect.

## 4. Discussion

Schizophrenia is increasingly recognized as a neurodevelopmental illness. Anomalies during pre- or perinatal brain development, as well as adult-onset brain changes before psychosis onset, have been proposed. Studies indicate that excessive dendritic elimination during adolescence may trigger the disease. Degenerative processes post-onset led to significant neuronal losses [20,21,22,23].

Computed tomography is widely available globally, and one of its clinical indications is psychosis [24,25]. Analyzing this rich data source provides a means to study and compare large and culturally diverse patient samples. By examining large samples, patient characteristics that may systematically affect results (e.g., gender, age, etc.) can be experimentally controlled, allowing the study of variables with a low prevalence. Additionally, evaluating multiple brain regions, either individually or in combination, can highlight morphological patterns, which might be more applicable than simple modifications in individual regions [26,27]. Routine cerebral CT could increase the probability of detecting anomalies [28] and brain morphology aspects with clinical implications [29]. This research strategy would be useful in generating hypotheses that can be tested using more precise imaging technology, such as MRI.

Our findings regarding ventricular widening and frontal lobe density align with the results of the Enigma Study. We observed a progressive increase in ventricular size from the control group to those experiencing their first psychotic episode and further enlargement in individuals diagnosed with schizophrenia [30].

In a 10-year study, brain scans were acquired from 15 individuals diagnosed with schizophrenia and 12 control participants during the initial assessment, and at follow-up evaluations after 4 and 10 years. The results indicated a significant difference in the long-term patterns of brain structural changes between those with schizophrenia and the control group. Notably, individuals with schizophrenia exhibited a significantly more pronounced enlargement of the ventricles over the specified time frame, a finding that is in line with the outcomes observed in our study [31].

Postmortem analyses conducted by Selemon et al. on the brains of individuals with schizophrenia, often carried out years after symptom onset, consistently unveil notable structural modifications. These investigations document increased cell-packing density, decreased neuronal soma cell size, and a decline in myelinated tracts within the brains of those affected by schizophrenia. Notably, there is a discernible 21% overall rise in neuronal density in the brains of individuals with schizophrenia when compared to those of normal controls. This increase is particularly noteworthy in specific cortical layers, specifically layers II, III, IV, and VI, underlining the intricate nature of neuronal alterations within distinct regions of the brain in individuals grappling with schizophrenia [32].

In a meta-analysis conducted by Petralia et al., a robust and disease-specific set of dysfunctional biological pathways characterizing schizophrenia patients is detailed. This involves the identification of increased neuronal densities in specific regions [33]. For instance, in Brodmann’s areas 9 and 46 of the prefrontal cortex, as well as Brodmann’s area 17 of the occipital cortex, the use of a quantitative analytic approach revealed heightened neuronal densities of 17%, 21%, and 10%, respectively. This increase was particularly evident in Brodmann’s area 9 in layers III-IV and in Brodmann’s area 46 in layers II-IV and VI. Moreover, the elevated densities were observed in both pyramidal and nonpyramidal neurons [32].

Currently, there are several new methods of imaging exploration. An example is the use of synaptic vesicle glycoprotein (SV2A) as a ligand for Positron Emission Tomography (PET) imaging in a variety of psychiatric disorders [34]. Given the proposed synaptic abnormalities in schizophrenia, this could become an important tool to investigate the physiopathology of schizophrenia in the near future [35]. Another new technique is NODDI, which evaluates the orientation, dispersion, and density of neurites that showed an altered microstructure of the gray matter in schizophrenia [36].

In conclusion, the information accumulated in recent neuroimaging studies has changed the notions related to the pathogenesis of schizophrenia. However, small sample sizes, variables occurring in study groups, and methodological limitations have resulted in numerous gaps in the current knowledge. Currently, the diagnostic value of imaging tools is limited to identifying organic brain pathologies in a small proportion of individuals with secondary psychoses. However, imaging techniques can be useful in predicting the disease’s direction of evolution. Imaging methods can also help monitor the effects of treatment, both pharmacological agents and cognitive therapy. Like neurological disorders, psychiatric disorders are more likely to be associated with considerable neural network dysfunction rather than discrete lesions [37]. For this reason, multivariate analysis of multimodal imaging data may provide better diagnostic and predictive value for schizophrenia and other psychotic disorders.

Like any study, our research has some limitations, including the utilization of CT imaging instead of MRI, and the relatively small number of cases. Important strengths of our study consist of the imaging assessment of patients with first-episode schizophrenia, exclusion of all organic or neurologic pathology cases, and exclusion of all substance abuse cases.

Further research on the evolution of brain alterations and their potential impact on the therapeutic response to antipsychotics is an ongoing project, the results of which will be published soon.

## 5. Conclusions

In summary, our comparative imaging analysis of patients with first-episode schizophrenia, schizophrenia diagnosed for more than 5 years, and control cases revealed distinct patterns in the frontal lobe and cerebral ventricles.

Concerning ventricular measurements, SCZ patients showed greater widening of the frontal horns and lateral ventricles compared to the FES group, with FES patients exhibiting wider ventricles compared to the control group. The third ventricle differed between SCZ and FES, while the fourth ventricle had similar averages in FES and SCZ, both higher than the control group.

Frontal lobe density, measured through cerebral CT scans, indicated higher density in the anterior and posterior portions of the cortex in SCZ patients compared to FES and the control group. Notably, the left posterior cortical region displayed the highest density in FES. The control group consistently showed the lowest densities.

These findings highlight distinctive neuroanatomical features across groups, providing insights into the structural differences associated with different stages of schizophrenia.

## Figures and Tables

**Table 1:** Demographics and clinical characteristics of the study population.

	FES	Schizophrenia		Control	*p* Value
Number of patients	51	49		50	-
Male gender (*n*, %)	28, 54.9%	24, 48.98%		34, 68%	0.55
Mean age (±SD)	26.35 ± 3.81	40.08 ± 2.89		34.60 ± 8.01	<0.0001
Onset age (±SD)	25.12 ± 3.75	24.96 ± 2.99		NA	0.81
Duration of illness (±SD)	1.20 ± 0.63	15.12 ± 4.04		NA	<0.0001
Antipsychotic treatment					
	FES	Mean dose (mg)	Chlorpromazine equivalent (mg)	SCZ	Mean dose (mg)
Amisulpride (*n*, %)	3, 5.88%	533.33	533.33	4, 8.16%	650
Aripiprazole (*n*, %)	6, 11.77%	25	333.33	3, 6.12%	20
Clozapine (*n*, %)	0	-	-	8, 16.33%	312.5
Olanzapine (*n*, %)	16, 31.37%	19.06	381.25	21, 42.86%	16.75
Quetiapine (*n*, %)	7, 13.73%	414.29	555.55	3, 6.12%	666.67
Paliperidone (*n*, %)	5, 9.80%	7.2	360	3, 6.12%	9
Risperidone (*n*, %)	14, 27.45%	3.93	196.43	7, 14.29%	5.14

**Table 2:** Statistical analysis of the frontal lobe structure using ANOVA.

	ANOVA Single Factor					
	Region	Factor	Average	F	F Critical	*p*-Value
Frontal right anterior	Cortical	SCZ	30.31	1.18	3.06	0.31
		FES	30.42			
		CONTROL	29.51			
	Subcortical	SCZ	29.47	3.15	3.06	<0.05
		FES	29.04			
		CONTROL	28.44			
Frontal left anterior	Cortical	SCZ	35.83	0.94	3.06	0.39
		FES	30.63			
		CONTROL	29.52			
	Subcortical	SCZ	29.55	9.21	3.06	<0.001
		FES	29.85			
		CONTROL	28.32			
Frontal right posterior	Cortical	SCZ	31.63	4.09	3.06	<0.05
		FES	31.78			
		CONTROL	30.41			
	Subcortical	SCZ	29.89	3.24	3.06	<0.05
		FES	29.79			
		CONTROL	29.04			
Frontal left posterior	Cortical	SCZ	31.77	5.89	3.06	<0.01
		FES	31.37			
		CONTROL	30.05			
	Subcortical	SCZ	30.04	7.03	3.06	<0.01
		FES	29.68			
		CONTROL	28.70			

**Table 3:** *T*-test for independent samples.

	*t*-Test for Independent Samples						
	Region	SCZ	FES	Control	Statistic	t Critical Two-Tail	*p*-Value
Frontal right anterior	Subcortical	29.47	29.04		1.05	1.66	0.29
		29.47		28.44	2.43	1.66	<0.05
			29.04	28.44	1.46	1.66	0.15
Frontal left anterior	Subcortical	29.55	29.85		−0.74	1.66	0.46
		29.55		28.32	3.44	1.66	<0.001
			29.85	28.32	4.17	1.66	<0.001
Frontal right posterior	Cortical	31.63	31.78		−0.28	1.98	0.78
		31.63		30.41	2.20	1.98	<0.05
			31.78	30.41	2.87	1.98	<0.01
	Subcortical	29.89	29.79		0.30	1.98	0.77
		29.89		29.04	2.26	1.98	<0.05
			29.79	29.04	2.01	1.98	<0.05
Frontal left posterior	Cortical	31.77	31.37		0.70	1.98	0.48
		31.77		30.05	3.37	1.98	<0.01
			31.37	30.05	2.69	1.98	<0.01
	Subcortical	30.04	29.68		0.90	1.98	0.37
		30.04		28.70	3.47	1.98	<0.001
			29.68	28.70	3.08	1.98	<0.01

**Table 4:** Bravais–Pearson correlation coefficient *r*.

	*t*-Test for Independent Samples					
	Region	SCZ	FES	Control	*p*-Value	r
Frontal right anterior	Subcortical	29.47	29.04		0.29	-
		29.47		28.44	<0.05	0.24
			29.04	28.44	0.15	-
Frontal left anterior	Subcortical	29.55	29.85		0.46	-
		29.55		28.32	<0.001	0.33
			29.85	28.32	<0.001	0.39
Frontal right posterior	Cortical	31.63	31.78		0.78	-
		31.63		30.41	<0.05	0.22
			31.78	30.41	<0.01	0.28
	Subcortical	29.89	29.79		0.77	-
		29.89		29.04	<0.05	0.22
			29.79	29.04	<0.05	0.20
Frontal left posterior	Cortical	31.77	31.37		0.48	-
		31.77		30.05	<0.01	0.32
			31.37	30.05	<0.01	0.26
	Subcortical	30.04	29.68		0.37	-
		30.04		28.70	<0.001	0.33
			29.68	28.70	<0.01	0.30

## Data Availability

Data were retrieved from the paper and electronic documents of the patients. The data sets used and/or analyzed during the current study are available from the corresponding author upon reasonable request.
